# Knowledge, attitude, and practice of health professionals for oxygen therapy working in South Gondar zone hospitals, 2021: multicenter cross-sectional study

**DOI:** 10.1186/s12913-022-08011-4

**Published:** 2022-05-04

**Authors:** Basazinew Chekol Demilew, Agegnehu Mekonen, Agazhe Aemro, Nakachew Sewnet, Banchiayehu Alebachew Hailu

**Affiliations:** 1 Department of Anesthesia, College of Health Sciences, Debre Tabor University, Debre Tabor, Ethiopia; 2 Department of Medical Nursing, College of Medicine and Health Science, University of Gondar, Gondar, Ethiopia; 3 Department Clinical Midwifery, College of Health Science, Debre Birhan University, Debre Birhan, Ethiopia; 4 Department of Public Health, College of Health Science, Debre Tabor University, Debre Tabor, Ethiopia

**Keywords:** Oxygen therapy, Oxygenation, Oxygen, Knowledge, Attitude, Practice

## Abstract

**Introduction:**

Therapeutic oxygen should be administered by competent healthcare providers who possess the required competencies of knowledge, skill, and judgment/abilities to make clinical decisions regarding the administration of oxygen. Therefore, this study aimed to assess the knowledge, attitude, and practice of health professionals towards oxygen therapy.

**Methods:**

A multicenter institutional-based cross-sectional study was conducted among 218 health professionals. The assessment was done with a total of 31 questions. After data cleanup analysis was done with SPSS software. Descriptive, chi-square test, bivariable and multivariable analysis were done accordingly. A *p*-value of ≤0.05 was considered to have a significant association with the outcome variables.

**Results:**

Among 218 participants, most of the participants (92.7%) were in the age range of less than 40 years old. Nurses were the most responding professions followed by physicians and midwifes. From the participants, around 54.6, 54.6, and 65.1% of respondents answered above the means score of knowledge, attitude and practice questions respectively. Getting training (AOR- 4.15, CI- 1.15-14.6), work experiences of less than 4 years (AOR- 2.54, 95%CI- (1.28-5.05), and availability of guidelines (AOR- 11.5, CI- 3.35-39.6) were significantly associated with knowledge level. Also work experience of fewer than 4 years (AOR- 3.41, 95%CI- (1.58-7.35) and presence of periodic maintenance and supply of oxygen therapy devices (AOR- 4.32, 95% CI- (1.44-12.9) were associated with practice level. Similarly, work experiences < 4 years (AOR- 8.6, 95%CI- (2.6-29) and getting training (AOR- 21.4, 95%CI-(2.7- 27.3) has a positive (direct) association with the level of attitude, and poor level of knowledge (AOR- 12.1, 95%CI (3.42-42.9) was contributed for negative attitude.

**Conclusion:**

This study concluded that 54.6, 54.6, and 65.1% of participants have a good level of knowledge, positive attitude, and good level of practice towards oxygen therapy respectively.

## Introduction

Oxygen is a commonly used drug in clinical settings like other medications, particularly for those patients who are at risk of respiratory failure. In recent times, several international bodies have advocated for the prescription of oxygen therapy in an attempt to reduce the risk of hypercapnia in vulnerable patients. Oxygen is essential to keep alive and work and in a certain way the cause of our death too [1, 2]. Supplemental oxygen is a well-established therapy for patients with chronic obstructive pulmonary diseases and severe resting hypoxemia, which is defined as a room air PaO_2_ ≤ 55 mmHg or ≤ 59 mmHg with signs of right-sided heart strain or polycythemia [1, 3–5].

It is required by all tissues to support cell metabolism; in acute illness, low tissue oxygenation (hypoxia) can occur due to a failure in any of the systems that deliver and circulate oxygen. Hypoxemia is an indication to start oxygen therapy; this can be a life-saving intervention, but given without appropriate assessment and ongoing evaluation can be detrimental to patients’ health [6–8].

To maintain adequate oxygen saturation for the patient who deteriorates, a high fraction of inspired oxygen (FIO_2_) may be required. For this reason, patients who need high FIO_2_ should receive senior clinician review and transfer to an area where there are appropriate numbers of competent staff able to provide more intensive monitoring and therapy [5, 7–10].

Competent healthcare providers who possess the required competencies (knowledge, skill, and judgment/abilities) are required to administer therapeutic oxygen and to make clinical decisions regarding the administration of oxygen [11].

A clear knowledge, attitude, and practice for oxygen therapy among health professionals who are managing patients at different patient care areas are mandatory. But it has gaps in real practice due to lack of oxygen therapy training and guideline, workload, inadequate supply of oxygen and delivery devices, and so on [1, 8, 12, 13]. National and possibly local oxygen therapy guidelines or hospital protocols are better to be developed and practiced. Oxygen supply and delivery devices should always be adequate and be used properly. Knowledge of oxygen therapy and the equipment used to deliver oxygen may also be barriers to optimal oxygen administration. Also, practice is one of the important factors that could contribute to oxygen desaturation in the clinical area [1, 10, 11]. As per our day to day clinical environment observation there is a real gap in knowledge, attitude and practice of oxygen therapy. Therefore this study aims to assess the knowledge, attitude and practice, and associated factors of health professionals towards oxygen therapy.

## Methods

### Study design, participants, area, and period

The multicenter institutional-based cross-sectional study was conducted on health professionals working in hospitals found in the South Gondar zone from January to May 2021. There are eight hospitals in South Gondar zone and from those we selected four hospitals to participate in the study. Debre Tabor referral hospital, Nefasmewucha primary hospital, Adiszemen primary hospital, and Mekaneeyesus primary hospital were included.

### Eligibility criteria

All health care professionals working in hospitals found in the south Gondar zone who were available in the study area during the study period were included by excluding those healthcare professionals on annual, maternity, and sick leave during the study period.

### Sampling technique and procedure

The single population proportion formula was used to determine the sample size. It was calculated by considering 95% CI, a 5% margin of error, and 50% as a proportion of good knowledge about oxygen therapy.

$$n=\frac{z^2\left(p\right)\left(1-p\right)}{d^2}\text{ }where;\text{ }n-\text{ }sample\text{ }size,p-\text{ }proportion\left(50\%\right).\text{ }d-\text{ }Precision\left(0.05\right)$$

$$\frac{\mathrm{n}={(1.96)}^20.5\left(1-0.5\right)\approx \mathbf{384}}{0.0{5}^2}$$

By applying a finite population correction formula since the total population in the study areas were around 510 health professionals, the final sample size will be;

*Nf* = *n*/(1 + *n*/*N*)***;*** Where, Nf = the minimum sample size***;*** *n =* sample size (384) & *N =* Total number of health professionals (510).

$$\mathrm{Nf}=\kern0.75em 384/\left(1+384/510\right)\approx 218$$

**Fig. 1:**
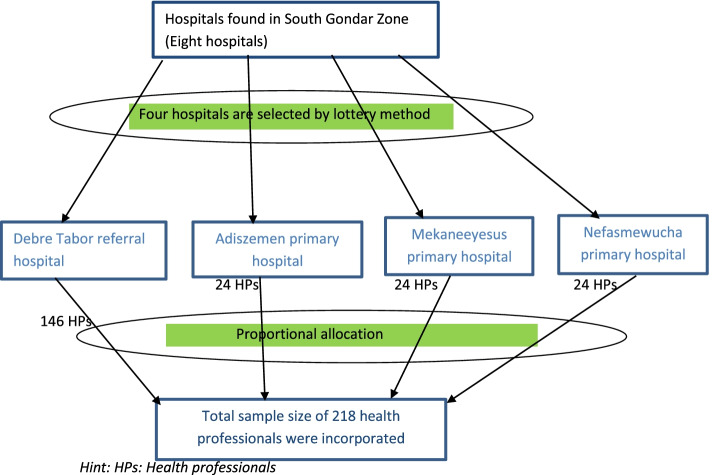
Flow chart showing the sampling procedure

Finally, a simple random sampling technique was employed to select hospitals and the study participants from the included hospitals. The total number of health professionals included in the study was proportional to the number of health professionals in each hospital and profession (Fig. 1).

### Study variables

Dependent variables: Knowledge (Good vs Poor), attitude (Positive vs Negative), and practice (Good vs Poor) level of health professionals were the outcome variables.

Independent variables: sociodemographic and work-related characteristics were the explanatory variables.

### Operational definition

#### Knowledge

Study participants who scored the mean score and above (≥68.7%) of the knowledge questions were considered to have good knowledge of oxygen therapy. Whereas those who scored below the mean score (< 68.7) were considered to have poor knowledge.

#### Attitude

Participants who answered ≥18.2% (mean score) of the attitude questions were considered to have a positive attitude towards oxygen therapy and < 18.2 were considered to have a negative attitude.

#### Practice

participants who scored 80.8% and above (mean score) of the practice questions were considered as having good practice and those who scored < 80.8% were considered as having poor practice.

### Data collection procedures

Two data collectors and a supervisor was employed in the data collection process. A self-administered questionnaire was prepared and used in the English language. The data collection tool was prepared from different literatures which were done on the same topic abroad [10, 14]. A total of 11 knowledge, 10 attitude, and 12 practice questions were used. The sum of correct responses for the 11 knowledge questions and 12 practice questions were computed and expressed in percentage to categorize whether study participants have good or poor knowledge and practice respectively. Similarly, Attitude was measured on a 5-point Likert scale and expressed in percentage to categorize whether study participants have a positive or negative attitude. Reliability of the tool was checked using reliability coefficient (Cronbach’s alpha) and was 0.7 for all knowledge, attitude and practice questions.

### Data quality assurance

To ensure the quality of data, training for the data collectors and the supervisor was given and a pre-test was done. The supervisor has checked the data collectors and the completeness of the tool every day during data collection time.

### Statistical analysis

Data were coded and entered into Epi-data version 4.2 and was exported to SPSS version 20 for statistical analysis. Categorical socio-demographic and work-related data were summarized by frequencies and percentages of occurrence. The chi-square test was used to compare the frequencies of respondents with categorical variables. Independent variables were analyzed using bivariate and multivariate logistic regression with the dependent variable. Variables with a *p*-value of ≤0.2 from the bivariate analysis were fitted to a multivariate logistic regression to check their association with dependent outcome. Adjusted Odds ratio with 95% confidence interval and the p-value of ≤0.05 was considered to have a significant association with the outcome variables.

### Ethical consideration

Ethical clearance and permission to conduct the research was obtained from the research and community service coordinator office of the college of health science, Debre Tabor University. Written informed consent was presented and obtained from each study participant according to the principles of the Helsinki declaration.

## Results

### Sociodemographic and work-related characteristics

Most of the participants (92.7%) were in the age range of less than 40 years old. Nurses were the most responding professions followed by physicians and midwifery department in the study. From the respondents, most health professionals are working in the ward, outpatient department, and emergency departments respectively (Table 1).

**Table 1:** Sociodemographic and work-related characteristics of health professionals working in south Gondar Zone hospitals, 2021

Variables	Category	Response n (%)
Age	< 40 years	202 (92.7)
	>/= 40 years	16 (7.3)
Sex	Male	126 (57.8)
	Female	92 (42.2)
Profession	Physician	57 (26.1)
	Anesthetists	13 (6)
	Nurses	100 (45.9)
	Midwifery	39 (17.9)
	IESO	9 (4.1)
Working area	Operation room	18 (8.3)
	ICU & NICU	24 (11)
	All Wards	97 (44.5)
	Recovery room	9 (4.1)
	Emergency	32 (14.7)
	All OPDs	38 (17.4)
Educational status	1st degree and below	182 (83.5)
	2nd degree and above	36 (16.5)
Experience	</= 4 years	83 (38.1)
	> 4 years	135 (61.9)H

Hint*: IESO* Integrated emergency surgery and obstetric*, ICU* Intensive care Unit*, OPD* Outpatient department

### Knowledge status of health professionals towards oxygen therapy

The participant’s responses for the knowledge questions range from a maximum score of 93.1% to a minimum score of 33.5% from all questions. The most correctly answered question was indications of oxygen therapy (203(93.1%)) followed by conditions that affect the pulse-oximetry reading (202(92.7%)). The least answered question was components of arterial blood gas analysis measures to detect respiratory problems (73(33.5%)) (Table 2).

**Table 2:** Health professionals of south Gondar zone hospitals who can answer the knowledge questions of this study correctly

Knowledge questions	Frequency	Percent
What is the advantage of oxygen administration?	190	87.2
What is the normal oxygen saturation at rest for an adult < 70 years old?	116	53.2
Which of the following is/are an indication of oxygen therapy?	203	93.1
What is the movement of air in to and out of the lung?	127	58.3
In respiratory physiology, what is the passive process of respiration?	174	79.8
What is the normal breathing rate for an adult in-breath per minute?	100	45.9
Which of the following conditions affect pulse-oximetry reading?	202	92.7
Supplemental oxygen is contraindicated for untreated pneumothorax	151	69.3
Non-rebreathing oxygen face mask with a reservoir bag is used to deliver higher oxygen concentration than a nasal prong.	173	79.4
The normal partial pressure of oxygen is	138	63.3
Which components of ABG measures detect the respiratory problem	73	33.5

Hint: *ABG* Arterial Blood Gas Analysis

### The attitude level of health professionals towards oxygen therapy

Most participants of the study agree on Oral &nasal hygiene and normal saline drops as necessary should be done when giving oxygen therapy (155(71.1%)) followed by the best practice to prevent dryness of the mucus membrane of the upper respiratory is humidification (151(69.3)) (Table 3).

**Table 3:** Responses to attitude questions by health professionals working at South Gondar Zone hospitals, Northwest Ethiopia, 2021(*N* = 218); n (%)

Attitude questions	Health professionals’ response to attitude questions of oxygen therapy				
	Strongly agree	Agree	Neutral	Disagree	Strongly disagree
Oxygen is a drug that should be given only when prescribed by a doctor or a registered nurse during an emergency.	75 (34.4)	76(34.9)	2(0.9)	64(29.4)	1(0.5)
Oral &nasal hygiene and normal saline drops as necessary should be done when giving oxygen therapy.	55(25.2)	155(71.1)	1(0.5)	7(3.2)	0
Intermittent oxygen therapy is more beneficial than continuous oxygen administration.	60(27.5)	144(66.1)	10(4.6)	3(1.4)	1(0.5)
The best practice to prevent dryness of the mucus membrane of the upper respiratory is humidification.	58(26.6)	151(69.3)	1(0.5)	6(2.8)	2(0.9)
Persons with severe lung disease need to maintain at the prescribed oxygen saturation range.	34(15.6)	70(32.1)	1(0.5)	57(26.1)	56(25.7)
Since oxygen is a drug its administration to the patient is not safe &very dangerous.	25(11.5)	30(13.8)	7(3.2)	77(35.3)	79(36.2)
A patient on oxygen therapy indicates that the patient is at the end stage of life.	8(3.7)	9(4.1)	4(1.8)	82(37.6)	115(52.8)
Since oxygen is a vasoactive substance, excessive supplementation leads to hyperoxia.	56(25.7)	141(64.7)	3(1.4)	11(5)	7(3.2)
High fractions of FiO2 cause lung damage.	71(32.6)	137(62.8)	3(1.4)	6(2.8)	1(0.5)
Oxygen saturation and delivery system should be recorded on the patient monitoring chart routinely.	75(34.4)	138(63.3)	1(0.5)	4(1.8)	

Hint: *FiO2* Fraction of Inspired Oxygen

### The practice of health professionals for oxygen therapy

Most of the respondents (98.6%) responded as they collect and check all necessary equipments before oxygen administration and follow the patient’s vital signs during oxygen administration equally. Whereas the least responses (38(17.4%)) respond to practice nasal cannula for the administration of oxygen with > 4 L/min even for patients having nasal polyps and nasal edema (Table 4).

**Table 4:** Responses to practice questions by health professionals working at South Gondar Zone hospitals, Northwest Ethiopia, 2021(*N =* 218); n (%)

Practice questions	Response - n (%)	
	Yes	No
Did you think pulse-oximetry is a good monitor for carbon-monoxide poisoned patients	190(87.2)	28(12.8)
Did you check the waveform and/or signal strength to accept the pulse oximetry reading	181(83)	37(17)
Does the blood pressure cuff on the hand with the pulse-oximetry probe affects the reading?	209(95.9)	8(3.7)
Did you attach a humidification device during oxygen administration?	174(79.8)	44(20.2)
Collection of water in the tubing during oxygen administration can affect oxygen administration?	207(95)	11(5)
Did you assess the patient regularly who is on oxygen therapy?	202(92.7)	16(7.3)
The nasal cannula is useful for administration of oxygen with > 4 L/min even for patients having nasal polyps and nasal edema?	38(17.4)	180(82.6)
Did you use a non-rebreathing oxygen mask to deliver a high percentage of oxygen (60 – 90%) for treatment emergencies?	144(66.1)	74(33.9)
Did you apply mouth care and/or water-based cream and/or petroleum jelly to prevent dryness and inflammation of the lips and nose during oxygen therapy?	180(82.6)	38(17.4)
Did you assess oxygen saturation before and during oxygen administration?	212(97.2)	6(2.8)
Did you collect and check all necessary equipments before oxygen administration?	215(98.6)	3(1.4)
Did you follow the patient’s vital signs during oxygen administration?	215(98.6)	3(1.4)

### Primary outcome variables

**Fig. 2:**
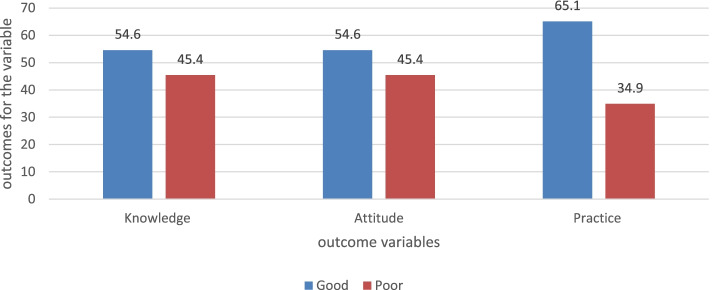
The overall results of the outcome variables for health professionals of South Gondar hospitals, Ethiopia, 2021

The mean score of the knowledge, attitude, and practice questions among the participants were 68.7, 18.2, and 80.8 respectively. Around 54.6% of the participants were answered above the mean value of knowledge and attitude questions. From the participants, 65.1% of answered above the mean value of the practice questions (Fig. 2).

### Factors associated with knowledge of health professionals

From all factors which were significantly associated with good knowledge in the bivariable logistic regression, only work experience, having training about oxygen therapy and availability of oxygen therapy guidelines or protocol in the working area were significantly associated with the good knowledge on the multivariable logistic regression. Accordingly, health professionals having less than or equal to four years of working experience are 2.54 times more likely to have good knowledge of oxygen therapy (Table 5).

**Table 5:** Factors affecting the knowledge level of health professionals who are working in South Gondar zone hospitals with bivariable and multivariable analysis

Factor variables	Knowledge				
	Good, n (%)	Poor, n (%)	COR, 95%CI	AOR, 95%CI	P
Age					
< 40	107 (49.1)	95 (43.6)	2.66,0.83-8.54	0.33,0.09-1.19	0.09
≥ 40	12 (5.5)	4 (1.8)	1		
Educational status					
1st degree &below	104 (47.7)	78 (35.8)	0.54,0.26-1.1	1.82,0.79-4.21	0.16
2nd degree &above	21 (9.6)	15 (6.9)	1		
Work Experience					
≤ 4 years	52 (23.9)	31 (14.2)	0.59,0.34-1.03	2.54,1.28-5.05	0.008*
> 4 years	68 (31.2)	67 (30.7)	1		
Oxygen therapy training					
Yes	22 (10.1)	30 (13.8)	1.92,1.02-3.6	4.15,1.15-14.6	0.03*
No	69 (31.7)	97 (44.5)	1		
Is there oxygen therapy guideline or protocols in your working area?					
Yes	43 (19.7)	25 (11.5)	0.59,0.33-1.07	11.5,3.35-39.6	0.001*
No	76 (34.9)	74 (33.9)	1		
Are you familiar with using devices of oxygen therapy?					
Yes	117 (53.7)	83 (38.1)	0.89,0.02-0.4	4.04,0.77-21.3	0.1
No	2 (0.9)	16 (7.3)	1		
Is there an adequate supply for the necessary materials of oxygen therapy?					
Yes	36 (16.5)	48 (22)	2.17,1.25-3.78	0.57,0.19-1.72	0.32
No	83 (38.1)	51 (23.4)	1		
Do you think workload affects oxygen therapy?					
Yes	110 (50.5)	82 (37.6)	0.4,0.17-0.93	1.92,0.68-5.42	0.22
No	9 (4.1)	17 (7.8)	1		

Hint: *AOR* Adjusted odds ratio, *COR* Crud odds ratio, *CI* Confidence interval, *implies significant association

### Factors affecting the practice of health professionals

After bivariable logistic regression was done factors having a *p*-value of 0.2 were fitted for multivariable logistic regression. Based on the multivariable logistic regression, work experience, workload, and periodic maintenance and supply of devices were found to be strongly associated with the good practice of health professionals. Based on the analysis, the presence of periodic maintenance and supply of oxygen therapy devices was 4.32 times more likely to contribute to good practice of oxygen therapy among the health professionals (Table 6).

**Table 6:** Factors affecting the practice level of health professionals who are working in South Gondar zone hospitals, 2021

Factors	Practice				
	Good, n (%)	Poor, n (%)	COR, 95%CI	AOR, 95%CI	P
Knowledge level					
Good	83(38.1)	36(16.5)	1		
Poor	59(27.1)	40(18.3)	1.56,0.89-2.74	0.72,0.35-1.46	0.36
Work Experience					
≤ 4	65 (29.8)	18 (8.3)	0.37,0.2-0.69	3.41,1.58-7.35	0.002*
> 4	77 (35.3)	58 (26.6)	1		
Oxygen therapy training					
Yes	40 (18.3)	12 (5.5)	0.48,0.23-0.98	1.2,0.36-3.97	0.77
No	102 (46.8)	64 (29.4)	1		
Is there oxygen therapy guideline or protocols in your working area?					
Yes	51 (23.4)	17 (7.8)	0.51,0.27-0.97	0.93,0.33-2.63	0.89
No	91 (41.7)	59 (27.1)	1		
Are you familiar with using devices of oxygen therapy?					
Yes	135 (61.9)	65 (29.8)	0.31,0.11-0.83	3,0.81-11.15	0.1
No	7 (3.2)	11 (5)	1		
Is there an adequate supply for the necessary materials of oxygen therapy?					
Yes	61(28)	23(10.6)	0.58,0.32-1.04	0.47,0.16-1.39	0.17
No	81(37.2)	53(24.3)	1		
Do you think workload affects oxygen therapy?					
Yes	133(61)	59(27.1)	0.24,0.1-0.56	5.99,2.1-17.15	0.001*
No	9(4.1)	17(7.8)	1		
Is there periodic maintenance and supply of oxygen therapy devices					
Yes	77(32.1)	22(10.1)	0.42,0.23-0.76	4.32,1.44-12.9	0.009*
No	72(33)	54(24.8)	1		
Is there an adequate supply of oxygen and delivery systems in your working area?					
Yes	46(21.1)	14(6.4)	0.47,0.24-0.93	1.9,0.78-4.64	0.16
No	96(44)	62(28.4)	1		

Hint: *AOR* Adjusted odds ratio, *COR* Crud odds ratio, *CI* Confidence interval; * = significantly associated

### Factors affecting the attitude of health professionals of oxygen therapy

Based on the multivariable logistic regression, knowledge level, educational status, work experience, and oxygen therapy training were significantly associated with the attitude of health professionals towards oxygen therapy. According to the analysis, the likely hood of having a positive attitude towards oxygen therapy was higher among health professionals having poor knowledge, being 1st degree /below, having ≤4 years of work experience and taking training about oxygen therapy (Table 7).

**Table 7:** Factors affecting the attitude level of health professionals who are working in South Gondar zone hospitals, 2021

Variables	Attitude				
	Positive,n(%)	Negative, n(%)	COR, 95%CI	AOR, 95%CI	P
Knowledge level					
Good	43(19.7)	76(34.9)	1		
Poor	76(34.9)	23(10.6)	0.17,0.09-0.31	12.1,3.42-42.9	0.001*
Educational status					
1st degree & below	91(41.7)	91(41.7)	3.5,1.5-8.1	0.22,0.07-0.71	0.01*
2nd degree & above	28(12.8)	8(3.7)	1		
Work Experience					
≤ 4	56(25.7)	27(12.4)	0.42,0.24-0.75	8.6,2.6-29	0.001*
> 4	63(28.9)	72(33)	1		
Oxygen therapy training					
Yes	49(22.5)	3(1.4)	0.05,0.01-0.15	21.4,2.7-27.3	0.004*
No	70(32.1)	96(44)	1		
Is there oxygen therapy guideline or protocols in your working area?					
Yes	47(21.6)	21(9.6)	0.41,0.23-0.76	0.2,0.03-1.3	0.09
No	72(33)	78(35.8)	1		
Are you familiar with using devices of oxygen therapy?					
Yes	106(48.6)	94(43.1)	2.3,0.79-6.71	1.2,0.23-6.2	0.83
No	13(6)	5(2.3)	1		
Is there an adequate supply for the necessary materials of oxygen therapy?					
Yes	77(35.3)	7(3.2)	0.04,0.02-0.1	30.8,4.77-198.9	
No	42(19.3)	92(42.2)	1		
Do you think workload affects oxygen therapy?					
Yes	100(45.9)	92(42.2)	2.5,1.0-6.2	0.32,0.07-1.35	0.12
No	19(8.7)	7(3.2)	1		
Is there periodic maintenance and supply of oxygen therapy devices					
Yes	77(35.3)	15(6.9)	0.1,0.05-0.19	1.1,0.26-4.39	0.93
No	42(19.3)	84(38.5)	1		

Hint: *AOR* Adjusted odds ratio, *COR* Crud odds ratio, *CI* Confidence interval; * = significantly associated

## Discussion

Having adequate knowledge, attitude, and skill of oxygen therapy among health professionals is required for quality patient’s care. Health professionals need to have such clinical management components and competencies for effective and quality patient care.

In this study around 68.7% of the participants had a good level of knowledge which is greater than the reports of studies done in Addis Ababa, Ethiopia (36.2%) (10, 15), Debre Tabor general hospital, Ethiopia (52%) [14], Harari public hospitals, Ethiopia (61.49%) [15], Beirut Lebanon (55.1%) [16], University Teaching Hospital of Kigali (23.2%) [17], and Eritrean hospitals (43.3%) [18]. The discrepancy of these reports might be due to differences in study participants, study settings, and sample.

Participants having work experience of ≤4 years are around twice more likely to have a good level of knowledge regarding oxygen therapy (AOR- 2.54, 95%CI- (1.28-5.05). This can be explained with health professionals having a short duration since graduation might have an acute knowledge and can answer the knowledge questions of this study. This is supported by the research done in Turkey which explained as knowledge score was more significantly associated with working experience of fewer than 5 years [19]. Health professionals getting training about oxygen therapy are around four times to have a good level of knowledge. The presence of working guidelines and protocol in the working area is significantly associated with a good level of knowledge. Similarly, in a study done in Riyadh oxygen therapy and availability of working guidelines were significantly associated with knowledge level [20]. But contrary to our findings, a study done in Turkey explained that professionals who got training on oxygen therapy don’t have an association with knowledge level [19].

In our study, 65.1% of participants were evaluated to have a good level of practice. This finding is greater than the reports of studies done in Debre Tabor, Ethiopia (33%) [14], Addis Ababa (43.4%) [21], and Eritrean study (45%) [18], the University Teaching Hospital of Kigali, Rwanda (32.3%) [17], Egypt (58%) [22]. Whereas it is less than study findings which was done in Iran (74.5%) [23]. This variation can be explained by different contributing factors like differences in study participants, sample size, and study period.

In this current study, participants with work experience of fewer than 5 years have a good level of practice for oxygen therapy (AOR- 3.41, 95%CI- (1.58-7.35). Also, health professionals working in the area which has periodic maintenance and supply of oxygen therapy devices are four times more likely to have a good level of practice (AOR- 4.32, 95% CI- (1.44-12.9). Another factor that has an association with practice level was expected to have workload. In agreement with this finding, a study done in Riyadh explained that getting training, adequate supply of devices, and workload were associated with the practice level of participants [20].

Regarding the attitude level of the current study, 54.6% of participants have a positive level of attitude towards oxygen therapy. This finding is in agreement with reports from the study done in Addis Ababa (53.3%) [21]. Factors that can contribute to the attitude level of participants were identified. Participants with a poor level of knowledge are more likely to have a negative attitude towards oxygen therapy (AOR- 12.1, 95%CI (3.42-42.9). Work experiences less than 4 years (AOR- 8.6, 95%CI- (2.6-29) and getting training of oxygen therapy (AOR- 21.4, 95%CI-(2.7- 27.3) has a positive (direct) association with the level of attitude.

## Conclusion & Recommendation

The overall findings of this study reveal that 54.6, 54.6, and 65.1% of participants have a good level of knowledge, positive attitude, and good level of practice towards oxygen therapy respectively. This implies around half of the participants had significant knowledge, attitude, and practice gap for oxygen therapy.

Based on the results of our study, we highly recommend to have working local guidelines and protocols in all areas of patient care for all types of professions. Also, workplace trainings are highly recommended for all types of professionals regarding oxygen therapy. Comparisons of knowledge, attitude, and practice of oxygen therapy across different working areas and professions are better to further investigations by other researchers.

### Limitations

This study was done with a small sample size. Variations of knowledge, attitude, and practice across different working areas and professions were not compared.

## Data Availability

All the necessary data will be found from the corresponding author for a reasonable request.

## Abbreviations

AOR: Adjusted Odds Ratio
ABG: Arterial Blood Gass
CI: Confidence Interval
COR: Crudd Odds Ratio
ICU: Intensive Care Unit
IESO: Integrated Emergency Surgery and Obstetrics
NICU: Neonatal Intensive Care Unit
OPD: Outpatient Department/Diagnosis
